# Supplementary material for: Melatonin Ameliorates Diquat-Induced Testicular Toxicity via Reducing Oxidative Stress, Inhibiting Apoptosis, and Maintaining the Integrity of Blood-Testis Barrier in Mice
Source: Toxics. 2023 Feb 8;11(2):160. doi: 10.3390/toxics11020160 (PMC9958747; doi:10.3390/toxics11020160)
Supplement: Supplementary file 1 [file toxics-11-00160-s001.zip › toxics-2176540-supplementary.pdf]

Melatonin Ameliorates Diquat-Induced Testicular Toxicity via Reducing Oxidative Stress, Inhibiting Apoptosis, and Maintaining the Integrity of Blood-Testis Barrier in Mice

Li Yang [1], Jianyong Cheng [1], Dejun Xu [2], Zelin Zhang [1], Rongmao Hua [3], Huali Chen [4], Jiaxin Duan [5], Xiaoya Li [1] and Qingwang Li [1,*]

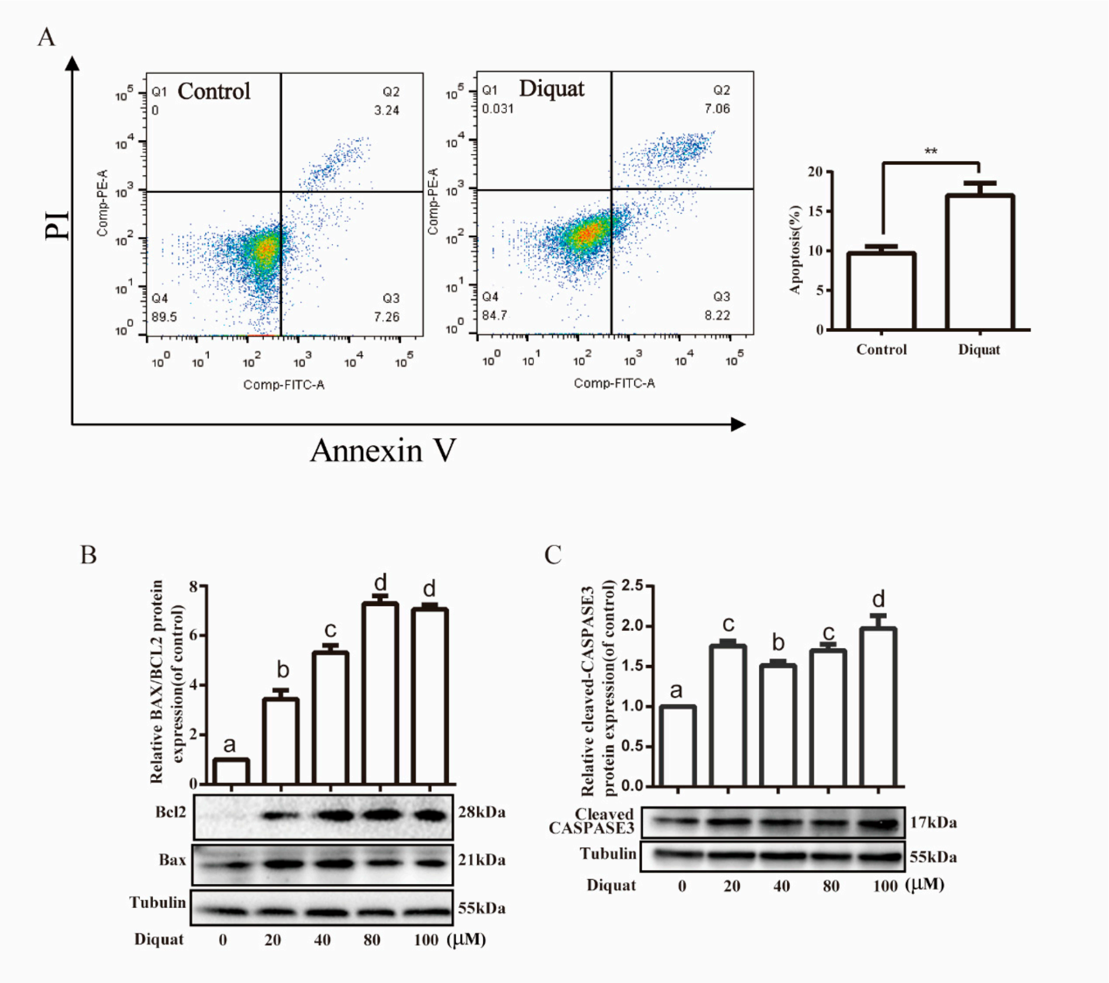

Figure S1. Diquat induced cell GC-1 spg apoptosis *in vitro*. (A) The cells were treated with diquat(80 μM) for 24 h. Apoptosis of the cells was detected by flow cytometry. (B,C) Representative western blotting and quantitative analysis of apoptosis-related markers protein Bax/Bcl2, Cleaved-Casepase3 proteins in different concentrations of diquat (0, 20, 40, 80 and 100μM). Data are presented as the mean ± SEM of at least three independent experiments. *$p < 0.05$, **$p < 0.01$. Values with different letters (a, b, c, d) indicate significant differences ($p < 0.05$).

Table S1. The primary antibodies information for western blot assay.

Antibody	Dilution	Immusourcer
Sod1	1:1000	Rabbit IgG
Gpx1	1:1000	Rabbit IgG
Bax	1:500	Rabbit IgG
Bcl2	1:500	Rabbit IgG
Cleaved Caspase3	1:500	Rabbit IgG
P53	1:500	Rabbit IgG
ZO-1	1:500	Rabbit IgG
Occludin	1:1000	Rabbit IgG
β -catenin	1:500	Rabbit IgG
Connexin43	1:1000	Rabbit IgG
Tubulin	1:2000	Rabbit IgG
